# The complete chloroplast genome sequence of *Cinnamomum kotoense*

**DOI:** 10.1080/23802359.2019.1703604

**Published:** 2019-12-18

**Authors:** Xiaolong Yuan, Yunqing Li, Yi Wang

**Affiliations:** Laboratory of Forest Plant Cultivation and Utilization, Yunnan Academy of Forestry, Kunming, People's Republic of China

**Keywords:** *Cinnamomum kotoense*, chloroplast, Illumina sequencing, phylogenetic analysis

## Abstract

The first complete chloroplast genome (cpDNA) sequence of *Cinnamomum kotoense* was determined from Illumina HiSeq pair-end sequencing data in this study. The cpDNA is 154,010 bp in length, contains a large single copy region (LSC) of 93,676 bp and a small single copy region (SSC) of 18,830 bp, which were separated by a pair of inverted repeats (IR) regions of 20,752 bp. The genome contains 127 genes, including 82 protein-coding genes, 8 ribosomal RNA genes, and 36 transfer RNA genes. The overall GC content of the whole genome is 39.2%, and the corresponding values of the LSC, SSC, and IR regions are 37.9%, 33.9%, and 44.3%, respectively. Further phylogenomic analysis showed that *C. kotoense* and *Cinnamomum bodinieri* clustered in a clade in Cinnamomum genus.

*Cinnamomum kotoense* Kanehira & Sasaki is an important small evergreen tree indigenous to Lanyu Island of Taiwan Province in China (Chen et al. 2006). *Cinnamomum kotoense* belongs to the species of genus *Cinnamomum* within the family Lauraceae, and is a kind of plant endowed with high pharmaceutical value and ornamental value (Cheng et al. 2010). The chemical constituents of *C. kotoense* possessed bioactive activities of anticancer, antioxidation and antibacterial (Chen et al. 2007; Kuo et al. 2008; Wang et al. 2010). In recent years, it has been widely cultivated for pot landscape plants in Taiwan, Yunnan, Guangxi, Guangdong and Fujian because of its bright evergreen leaves and beautiful tree shape (Yi et al. 2017). However, there has not found yet genomic report about *C. kotoense*.

Herein, we reported and characterized the complete *C. kotoense* plastid genome (MN698964). One *C. kotoense* individual (specimen number: 5309270232) was collected from Kunming botanical garden, Kunming, Yunnan Province of China (25°14′16″N, 102°75′13″E). The specimen is stored at Yunnan Academy of Forestry Herbarium, Kunming, China and the accession number is YAFH0012766. DNA was extracted from its fresh leaves using DNA Plantzol Reagent (Invitrogen, Carlsbad, CA).

Paired-end reads were sequenced by using Illumina HiSeq system (Illumina, San Diego, CA). In total, about 21.2 million high-quality clean reads were generated with adaptors trimmed. Aligning, assembly, and annotation were conducted by CLC de novo assembler (CLC Bio, Aarhus, Denmark), BLAST, GeSeq (Tillich et al. 2017), and GENEIOUS v 11.0.5 (Biomatters Ltd, Auckland, New Zealand). To confirm the phylogenetic position of *C. kotoense*, other five species of *Cinnamomum* genus from NCBI were aligned using MAFFT v.7 (Katoh and Standley 2013). The Auto algorithm in the MAFFT alignment software was used to align the six complete genome sequences and the G-INS-i algorithm was used to align the partial complex sequences. The maximum-likelihood (ML) bootstrap analysis was conducted using RAxML (Stamatakis 2006); bootstrap probability values were calculated from 1000 replicates. *Alseodaphne gracilis* (MG407593) and *Alseodaphne huanglianshanensis* (MG407594) were served as the out-group.

The complete *C. kotoense* plastid genome is a circular DNA molecule with the length of 154,010 bp, contains a large single copy region (LSC) of 93,676 bp and a small single copy region (SSC) of 18,830 bp, which were separated by a pair of inverted repeats (IR) regions of 20,752 bp. The overall GC content of the whole genome is 39.2%, and the corresponding values of the LSC, SSC, and IR regions are 37.9%, 33.9%, and 44.3%, respectively. The plastid genome contained 127 genes, including 82 protein-coding genes, 8 ribosomal RNA genes, and 36 transfer RNA genes. Phylogenetic analysis showed that *C. kotoense* and *Cinnamomum bodinieri* clustered in a unique clade in *Cinnamomum* genus (Figure 1). The determination of the complete plastid genome sequences provided new molecular data to illuminate the *Cinnamomum* genus evolution.

**Figure 1.**
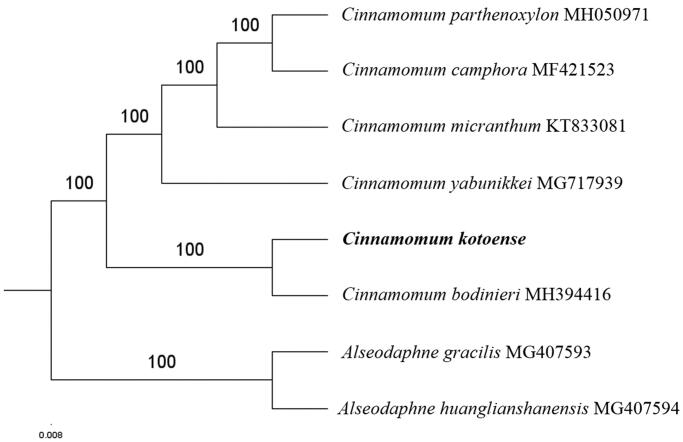
The maximum-likelihood tree based on the six chloroplast genomes of *Cinnamomum* genus. The bootstrap value based on 1000 replicates is shown on each node.

## References

[CIT0001] Chen CH, Lo WL, Liu YC, Chen CY. 2006. Chemical and cytotoxic constituents from the leaves of *Cinnamomum kotoense*. J Nat Prod. 69(6):927–933. 16792412 10.1021/np060107l

[CIT0002] Chen CY, Hsu YL, Chen YY, Hung JY, Huang MS, Kuo PL. 2007. Isokotomolide A, a new butanolide extracted from the leaves of *Cinnamomum kotoense*, arrests cell cycle progression and induces apoptosis through the induction of p53/p21 and the initiation of mitochondrial system in human non-small cell lung cancer A549 cells. Eur J Pharmacol. 574(2–3):94–102. 17707793 10.1016/j.ejphar.2007.07.028

[CIT0003] Cheng KC, Hsueh MC, Chang HC, Lee AYL, Wang HM, Chen CY. 2010. Antioxidants from the leaves of *Cinnamomum kotoense*. Nat Prod Commun. 5(6):911–912. 20614822

[CIT0004] Katoh K, Standley DM. 2013. MAFFT multiple sequence alignment software version 7: improvements in performance and usability. Mol Biol Evol. 30(4):772–780. 23329690 10.1093/molbev/mst010 PMC3603318

[CIT0005] Kuo PL, Chen CY, Tzeng TF, Lin CC, Hsu YL. 2008. Involvement of reactive oxygen species/c-Jun NH2-terminal kinase pathway in kotomolide A induces apoptosis in human breast cancer cells. Toxicol Appl Pharm. 229(2):215–226. 10.1016/j.taap.2008.01.034 18374381

[CIT0006] Stamatakis A. 2006. RAxML-VI-HPC: maximum likelihood-based phylogenetic analyses with thousands of taxa and mixed models. Bioinformatics. 22(21):2688–2690. 16928733 10.1093/bioinformatics/btl446

[CIT0007] Tillich M, Lehwark P, Pellizzer T, Ulbricht-Jones ES, Fischer A, Bock R, Greiner S. 2017. GeSeq-versatile and accurate annotation of organelle genomes. Nucleic Acids Res. 45(W1):W6–W11. 28486635 10.1093/nar/gkx391 PMC5570176

[CIT0008] Wang HM, Cheng KC, Lin CJ, Hsu SW, Fang WC, Hsu TF, Chiu CC, Chang HW, Hsu CH, Lee A. 2010. Obtusilactone A and (-)–sesamin induce apoptosis in human lung cancer cells by inhibiting mitochondrial Lon protease and activating DNA damage checkpoints. Cancer Sci. 101(12):2612–2620. 21077998 10.1111/j.1349-7006.2010.01701.x PMC11158771

[CIT0009] Yi RH, Liang YL, Xu XL, Xu YJ, He MZ, Chen J. 2017. Pathogen identification and biological characteristics of Colletotrichum aotearoa causing anthracnose diseases on *Cinnamomum kotoense*. J Central South Univ Forest Technol. 37(3):24–31.

